# A complex systems lens can help us understand drivers of emerging challenges in work and health

**DOI:** 10.5271/sjweh.4178

**Published:** 2024-09-01

**Authors:** Naja Hulvej Rod, Bertina Kreshpaj, Karien Stronks

**Affiliations:** 1 Copenhagen Health Complexity Center, Department of Public Health, University of Copenhagen, Copenhagen.; 2 Department of Public and Occupational Health, Amsterdam University Medical Centers, University of Amsterdam, The Netherlands.

## Emergent health challenges related to work

Work is not only central to population health but is also a significant driver of social inequality in health (1). In a recent Lancet series on work and health, the authors outlined six emergent challenges concerning work: the impact of technology, the intersection of work with sociodemographic health determinants, migrant work, precarious employment, long working hours, and climate change (1). The authors of the *Lancet* series also presented recommendations for future research, advocating for the utilization of mixed-methods, innovative analytical approaches (eg, causal modeling), realist evaluation, and interdisciplinary collaboration. Although each of these approaches are highly relevant, their integrated application was only vaguely outlined.

We believe that each of these work and health challenges show features of complex adaptive systems. They are multifaceted, constantly evolving, and emerge from our complex and disordered real world, which is often characterized by interactions, non-linearity, interference, feedback loops, and adaptation. Consequently, future research on work and health may benefit from adopting a complex systems perspective to obtain a comprehensive understanding of the drivers of these challenges (2–4).

We have recently developed an interdisciplinary framework for knowledge production aimed at understanding complex health issues within the domain of public health, rooted in complex systems theory (5). This framework can serve to organize our thinking, formulate research questions, and integrate methodologies related to each of these six work and health challenges.

Briefly outlined, the Health Complexity framework relates to three core dimensions in which complex health issues may be conceptualized: patterns, mechanisms, and dynamics (5).

*Patterns:* Looking for specific patterns of disease or risk factors allows us to empirically identify health issues that emerge from the mechanisms and dynamics of the underlying systems, eventually allowing us to discover vulnerable subgroups, and thereby set boundaries for targeted interventions.
*Mechanisms:* Understanding the core mechanisms that give rise to these emergent health patterns and how they are connected across scales through interactions and interference can help us identify potential leverage points for intervention.
*Dynamics:* Building evidence on the dynamics that make patterns and mechanisms change over time will allow us to identify vicious circles associated with particularly high morbidity.

Between them, these dimensions cover seven key features of complex systems (emergence, interactions, non-linearity, interference, feedback loops, adaptation, and evolution), which we have highlighted as central to public health. The Health Complexity framework builds upon the ideas of methodological pluralism (6–8) and is intended to be an overarching framework for interdisciplinary and collaborative research on complex health issues, also in the field of work and health. As an illustration, we will outline the elements needed to examine one of these challenges – precarious employment – through a complex systems lens, particularly highlighting how this approach influences the way we phrase research questions on health problems that do justice to the complexity of the real world.

## Precarious employment viewed through a complex systems lens

**Figure 1:**
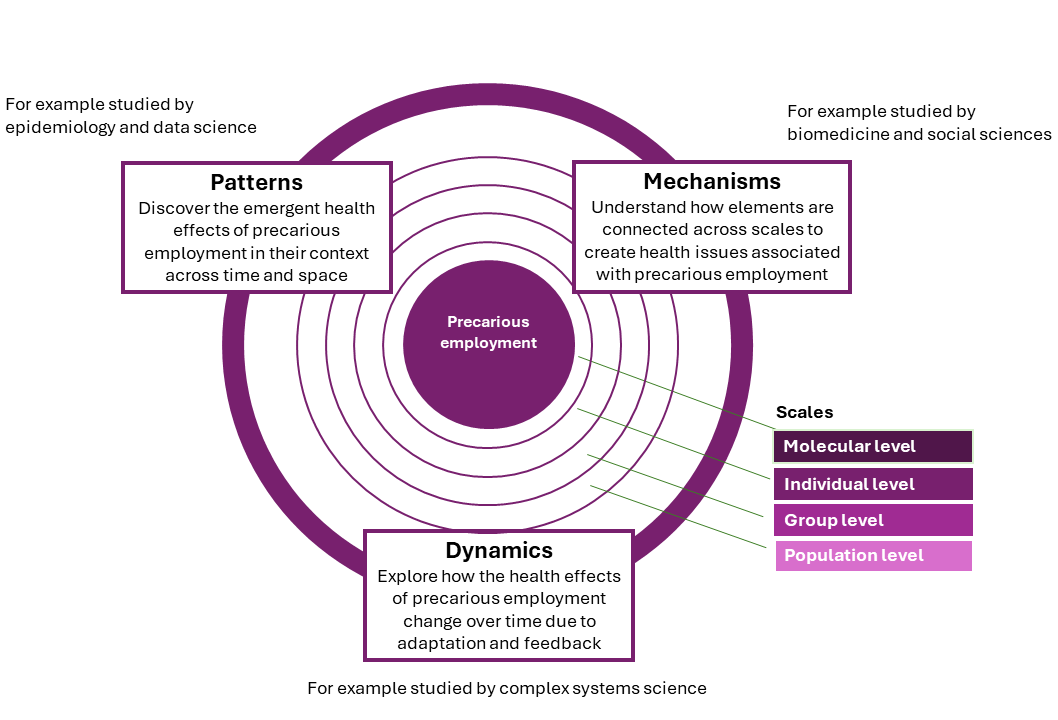
Health Complexity Framework for Precarious Employment and Health. Conceptual figure adapted from Rod et al. Complexity in Epidemiology and Public Health. Epidemiology 2023 (5).

With globalization and technological advancements, there has been a shift towards a gig economy. This has led to an increase in temporary, part-time, and freelance work, which often lacks stability and benefits. Precarious employment specifically refers to such work characterized by employment insecurity, income inadequacy, and lack of rights and protection (9). The lack of stability and benefits associated with precarious employment combined with poor working conditions have been shown to have negative effects on physical and mental health (10–13). Workers may experience higher levels of stress, depression, and other health problems due to financial insecurity and lack of access to healthcare, which collectively may be an important driver of health inequality and of health decline. In a life course perspective, there may also be important feedback mechanisms exacerbating such inequality, with poor health not only being a consequence of precarious employment, but workers with poor health may be more likely to be excluded from stable work (14). Overall, the increasing prevalence of precarious employment represents a substantial challenge for public health, which can be seen as a sort of byproduct of larger societal trends. We believe that employing a complex systems lens can help us generate relevant scientific knowledge about the fundamental drivers of this problem. This essentially entails three interlinked steps organized around the three core dimensions of the Health Complexity Framework (figure 1).

*Patterns:* As a first step, we need to zoom out and understand the health effects associated with emergent patterns of precarious employment in their context across time and space, asking questions such as:

How does precarious employment change over time, and how does this changing pattern affect population health?
Are there certain population groups, defined, eg, by socioeconomic status, age, occupation, migrant status, or geographical regions who experience more adverse health effects by precarious employment than others?

Systematically evaluating health patterns associated with precariousness can help us define boundaries for targeted prevention. Employing classical epidemiological surveillance methods alongside data science techniques for uncovering patterns within multidimensional large-scale datasets serves as key examples of such pattern identification.

*Mechanisms:* As a second step, we need to understand what mechanisms underlying the health effects of precariousness and how elements of these mechanisms are connected across scales, from cells to society, asking questions such as:

How do mechanisms interact across biological, behavioral, social, and societal scales to create the rising public health problems associated with precarious employment?
Does precarious employment and its associated health problems cluster and spread across social networks and/or across occupational and economic sectors?

Systematically evaluating the interconnectedness between mechanisms and individuals across various scales can help us identify leverage points for intervention. Whereas biomedical studies can contribute to uncovering the biological mechanisms linked to precarious employment, such as the embodiment of stress (15), the social sciences may offer profound insights into macro-scale mechanisms involving political, economic, and social structures.

*Dynamics:* As a third step, we need to explore how the health effects of precarious employment change over time due to dynamic processes like adaptation and feedback, asking questions such as:

How do national political and social contexts adapt to historical changes in the labor market including the increase in precarious employment, and what is the impact of this adaptation when it comes to how and to what extent precarious employment can affect the health of individuals and populations?
Is there a reinforcing feedback mechanism between social disadvantage, precarious employment, and health? This mechanism could create a vicious circle—for example, social disadvantage increasing the likelihood of precarious employment, which then leads to health consequences that may further reinforce social disadvantage.

Systematically assessing such dynamism can help us intervene on vicious circles that generate excessive burdens of disease in specific population groups. Systems methodology, including formal conceptual model building and computational simulations, are essential in creating such evidence.

Integrating interdisciplinary knowledge across these dimensions will provide a systematic and comprehensive understanding of the patterns of precarious employment and health, the underlying connected mechanisms generating these patterns, and the dynamics that makes them change over time. Some dimensions, like the patterns of precarious employment and health, may already be well-researched, while other dimensions such as dynamics require further investigation. We argue that it is essential to systematically explore all these dimensions to comprehensively understand a complex issue. Leaving out one of these core dimensions may leave blind spots that will render our understanding of precarious employment and health incomplete and thereby impact the efficiency of future interventions.

In this editorial, we have focused on how to phrase research questions when applying a complex systems lens on precarious employment and health. This clearly needs to be matched by the integration of an interdisciplinary set of methods and data. An overview of such methods and data can be found in Rod et al (5).

## Are we at the brink of a ‘complexity turn’ in public health?

We believe that we are witnessing a shift in public health away from the traditional model of evidence, which primarily focused on empirically testing predefined hypotheses of single exposures and outcomes. Instead, there is a growing recognition of public health issues as complex, involving the complex interactions of biological, social, psychological, economic, and other processes across various levels and time scales (2–5, 16–20). These dynamics may show nonlinearity and adaptability. This paradigm shift is particularly important to our understanding of the relationship between work and health, including the emergent challenges outlined in the *Lancet* series, where contextual factors and interactions across micro-, meso- and macro-levels emerge as main drivers of dynamic change in employment condition.

Formalizing this turn towards complexity in public health requires not only a realignment of our research questions as outlined for precarious employment above, but also necessitates the integration of traditional epidemiological methods with systems methodologies, such as computational simulation modeling (3, 18). Furthermore, it calls for sustained support for interdisciplinary collaboration and substantial investment in a diverse array of data types. These include multi-scale data, spatial data, time-series data, life-course data, network data, and multi-generational data, among others.

This shift in our understanding of public health also impacts our approach to evidence synthesis. Traditionally, evidence synthesis has been relatively straightforward, typically summarized in systematic reviews or meta-analyses focusing on single isolated risk factors. However, with a complex systems perspective, we must transition towards a dynamic evidence synthesis framework. This approach involves an ongoing process of data-driven discoveries, hypothesis testing, and theory building. By adopting this dynamic approach, we can effectively synthesize evidence on complex research questions while continuously assessing which dimensions remain unresolved and understudied. These unresolved or understudied aspects should serve as guiding principles for future studies and research programs, also on work and health.

## References

[1] Frank J, Mustard C, Smith P, Siddiqi A, Cheng Y, Burdorf A et al. Work as a social determinant of health in high-income countries: past, present, and future. Lancet 2023 Oct;402(10410):1357–67. 10.1016/S0140-6736(23)00871-1 37838441

[2] Rutter H, Savona N, Glonti K, Bibby J, Cummins S, Finegood DT et al. The need for a complex systems model of evidence for public health. Lancet 2017 Dec;390(10112):2602–4. 10.1016/S0140-6736(17)31267-9 28622953

[3] Stronks K, Crielaard L, Rod NH. Systems Approaches to Health Research and Prevention. In: Ahrens W, Pigeot I, eds. Handbook of Epidemiology. New York, NY: Springer, New York, NY, 2024: 1-29. 10.1007/978-1-4614-6625-3_70-1 10.1007/978-1-4614-6625-3_70-1

[4] Diez Roux AV. Complex systems thinking and current impasses in health disparities research. Am J Public Health 2011 Sep;101(9):1627–34. 10.2105/AJPH.2011.300149 21778505 PMC3154209

[5] Rod NH, Broadbent A, Rod MH, Russo F, Arah OA, Stronks K. Complexity in Epidemiology and Public Health. Addressing Complex Health Problems Through a Mix of Epidemiologic Methods and Data. Epidemiology 2023 Jul;34(4):505–14. 10.1097/EDE.0000000000001612 37042967 PMC10712344

[6] Ogilvie D, Bauman A, Foley L, Guell C, Humphreys D, Panter J. Making sense of the evidence in population health intervention research: building a dry stone wall. BMJ Glob Health 2020 Dec;5(12):e004017. 10.1136/bmjgh-2020-004017 33298470 PMC7733100

[7] Vandenbroucke JP, Broadbent A, Pearce N. Causality and causal inference in epidemiology: the need for a pluralistic approach. Int J Epidemiol 2016 Dec;45(6):1776–86. 10.1093/ije/dyv341 26800751 PMC5841832

[8] Illari PM, Russo F. Causality: philosophical theory meets scientific practice. Oxford: Oxford University Press, 2014.

[9] Kreshpaj B, Orellana C, Burström B, Davis L, Hemmingsson T, Johansson G et al. What is precarious employment? A systematic review of definitions and operationalizations from quantitative and qualitative studies. Scand J Work Environ Health 2020 May;46(3):235–47. 10.5271/sjweh.3875 31901944

[10] Matilla-Santander N, Muntaner C, Kreshpaj B, Gunn V, Jonsson J, Kokkinen L et al. Trajectories of precarious employment and the risk of myocardial infarction and stroke among middle-aged workers in Sweden: A register-based cohort study. Lancet Reg Health Eur 2022 Feb;15:100314. 10.1016/j.lanepe.2022.100314 35169764 PMC8829810

[11] Matilla-Santander N, Matthews AA, Gunn V, Muntaner C, Kreshpaj B, Wegman DH et al. Causal effect of shifting from precarious to standard employment on all-cause mortality in Sweden: an emulation of a target trial. J Epidemiol Community Health 2023 Nov;77(11):736–43. 10.1136/jech-2023-220734 37620008 PMC10579471

[12] Jonsson J, Muntaner C, Bodin T, Alderling M, Rebeka R, Burström B et al. Low-quality employment trajectories and risk of common mental disorders, substance use disorders and suicide attempt: a longitudinal study of the Swedish workforce. Scand J Work Environ Health 2021 Oct;47(7):509–20. 10.5271/sjweh.3978 34397098 PMC8504160

[13] Rönnblad T, Grönholm E, Jonsson J, Koranyi I, Orellana C, Kreshpaj B et al. Precarious employment and mental health: a systematic review and meta-analysis of longitudinal studies. Scand J Work Environ Health 2019 Sep;45(5):429–43. 10.5271/sjweh.3797 31165899

[14] Junna L, Moustgaard H, Martikainen P. Health-related selection into employment among the unemployed. BMC Public Health 2022 Apr;22(1):657. 10.1186/s12889-022-13023-0 35382786 PMC8985275

[15] McEwen BS. Neurobiological and Systemic Effects of Chronic Stress. Chronic Stress (Thousand Oaks) 2017;1:2470547017692328. 10.1177/2470547017692328 28856337 PMC5573220

[16] Page SE, Zelner J. Population Health as a Complex Adaptive System of Systems. In: Apostolopoulos Y, Lich KH, Lemke MK, eds. Complex Systems and Population Health, 1st edn. New York: Oxford University Press, 2020: 33-44. 10.1093/oso/9780190880743.003.0003 10.1093/oso/9780190880743.003.0003

[17] Rod MH, Rod NH, Russo F, Klinker CD, Reis R, Stronks K. Promoting the health of vulnerable populations: three steps towards a systems-based re-orientation of public health intervention research. Health Place 2023 Mar;80:102984. 10.1016/j.healthplace.2023.102984 36773380

[18] El-Sayed AM, Galea S. Systems Science and Population Health. New York: Oxford University Press, 2017. 10.1093/acprof:oso/9780190492397.003.0017 10.1093/acprof:oso/9780190492397.003.0017

[19] Luna Pinzon A, Stronks K, Dijkstra C, Renders C, Altenburg T, den Hertog K et al. The ENCOMPASS framework: a practical guide for the evaluation of public health programmes in complex adaptive systems. Int J Behav Nutr Phys Act 2022 Mar;19(1):33. 10.1186/s12966-022-01267-3 35346233 PMC8962023

[20] Stronks K, Nicolaou M. Embracing complexity in social epidemiology. Lancet Public Health 2018 Aug;3(8):e352–3. 10.1016/S2468-2667(18)30137-3 30030109

